# Negative control exposure studies in the presence of measurement error: implications for attempted effect estimate calibration

**DOI:** 10.1093/ije/dyx213

**Published:** 2017-10-27

**Authors:** Eleanor Sanderson, Corrie Macdonald-Wallis, George Davey Smith

**Affiliations:** MRC Integrative Epidemiology Unit, University of Bristol, Bristol, UK

**Keywords:** Negative control exposure, measurement error, unobserved confounding

## Abstract

**Background:**

Negative control exposure studies are increasingly being used in epidemiological studies to strengthen causal inference regarding an exposure-outcome association when unobserved confounding is thought to be present. Negative control exposure studies contrast the magnitude of association of the negative control, which has no causal effect on the outcome but is associated with the unmeasured confounders in the same way as the exposure, with the magnitude of the association of the exposure with the outcome. A markedly larger effect of the exposure on the outcome than the negative control on the outcome strengthens inference that the exposure has a causal effect on the outcome.

**Methods:**

We investigate the effect of measurement error in the exposure and negative control variables on the results obtained from a negative control exposure study. We do this in models with continuous and binary exposure and negative control variables using analysis of the bias of the estimated coefficients and Monte Carlo simulations.

**Results:**

Our results show that measurement error in either the exposure or negative control variables can bias the estimated results from the negative control exposure study.

**Conclusions:**

Measurement error is common in the variables used in epidemiological studies; these results show that negative control exposure studies cannot be used to precisely determine the size of the effect of the exposure variable, or adequately adjust for unobserved confounding; however, they can be used as part of a body of evidence to aid inference as to whether a causal effect of the exposure on the outcome is present.

**Key Messages**

- Negative control exposure studies can contribute to a triangulation of evidence to estimate the effect of an exposure on an outcome in the presence of suspected unobserved confounding.
- In the presence of measurement error, negative control exposure studies do not give a reliable estimate of the causal effect of the exposure on the outcome.
- Negative control exposure studies should not be used in effect estimate calibration to obtain an estimate of the causal effect of an exposure on an outcome.

## Introduction

In the presence of unobserved confounding, the causal effect of an exposure on an outcome of interest cannot simply be determined by regressing the outcome on the exposure. A method that has often been used to detect confounding and help assessment of whether a causal relationship exists between an exposure and an outcome is a negative control exposure study.^1^^,^^2^ Negative control exposure studies compare the association between an exposure of interest and an outcome with the association between a control variable and the same outcome. The control variable is chosen to be a variable that has no effect on the outcome of interest but is subject to the same unobserved confounding as the exposure of interest. Therefore, any association observed between the negative control and the outcome will be due to confounding in the model.^3^ If the association observed between the exposure of interest and the outcome is markedly larger than the association between the negative control and the outcome, then this can add to the evidence that the exposure of interest does have a causal effect on the outcome, and can feed into a triangulation of evidence on the causal effect of exposure on the outcome from a wide range of sources.^4–6^ If triangulation of the results from studies that suffer from different types of potential bias point to the same relationship between the exposure and the outcome, then this provides evidence for a causal association between the exposure and outcome. Fuller discussion of the use of negative controls in epidemiology is available elsewhere.^1^^,^^2^^,^^4^^,^^7–13^

One area where negative controls have often been used in epidemiology is to determine the effect of intrauterine exposure on later outcomes by comparing the association of a maternal exposure during pregnancy with the outcome of interest, with the association of the paternal exposure with the same outcome.^2^^,^^7^^,^^14^ If an intrauterine effect of the mother’s exposure on the child is present, the association of the maternal exposure with the outcome is expected to be larger than the association of the equivalent paternal exposure. Examples of studies where this type of negative control has been used include the effect of maternal and paternal smoking on offspring outcomes,^7^^,^^15–25^ the effect of maternal and paternal body mass index (BMI) on later offspring BMI,^26–33^ the effect of maternal and paternal diabetes on predisposition to diabetes in offspring,^34^ the effect of maternal and paternal energy intake on later offspring dietary intake^35^ and the effect of maternal and paternal BMI on offspring autism spectrum disorder.^36^

Another example of where negative control exposure studies have been used to evaluate the effect of intrauterine exposure, without using the paternal exposure as a control, is examining the association of mothers taking folic acid supplements in pregnancy compared with the negative control of taking other supplements, with autism^37^ and language development delays^38^ in their children. Other examples include the association of maternal smoking during pregnancy compared with maternal smoking after pregnancy, with offspring respiratory outcomes,^39^ the effect of maternal alcohol consumption during pregnancy compared with the negative control of maternal alcohol consumption before pregnancy, on offspring ADHD symptoms^40^ and the effect of exposure to air pollution before, during and after pregnancy, on autism spectrum disorder in offspring.^41^ A list of examples of negative control exposure studies is given in Table 1.

**Table 1:** Selected examples of studies which have used negative control exposure methods

Exposure	Negative control exposure	Outcome(s)
Maternal smoking	Paternal smoking	Offspring outcomes: Inattention/hyperactivity^15^^,^^20^ Obesity/adiposity^16^^,^^22–24^
		Blood pressure^17^
		Gestational diabetes^21^
		ADHD symptoms^19^
		Cognitive development^18^
		Offspring psychotic symptoms^46^
Maternal psychosocial stress	Paternal psychosocial stress	Offspring vascular function^54^
Maternal smoking during pregnancy	Maternal smoking after pregnancy	Offspring respiratory outcomes^39^
		Offspring psychotic symptoms^46^
Maternal alcohol consumption during pregnancy	Maternal alcohol consumption before pregnancy	Offspring ADHD symptoms^40^
Maternal BMI/obesity	Paternal BMI	Offspring BMI/adiposity^26–33^
		Offspring cognitive and psychomotor development^55^
Length of pre-birth inter-pregnancy interval	Length of post-birth inter-pregnancy interval	Risk of schizophrenia in the offspring^56^
Folic acid supplements in pregnancy	Other supplements in pregnancy	Autism spectrum disorders^37^
		Language development delays^38^
Prescription for trimethoprim 1–3 months before pregnancy	Prescription for trimethoprim 13–15 months before pregnancy	Offspring congenital malformation^57^
Air pollutant exposure during pregnancy	Air pollutant exposure before and after pregnancy	Offspring autism spectrum disorder^41^
Exposure to childhood infections	Hospital attendance for broken bones	Multiple sclerosis later in life^58^
Adherence to prescribed statins and beta blockers	Adherence to other prescribed medication	Long-term mortality after acute myocardial infarction^59^
Vaccination during flu season	Vaccination outside flu season	Mortality and hospitalization from flu^60^
Swimmers’ exposure to bacteria in water	Non-swimmers	Gastrointestinal illnesses after an increase in bacteria levels in water^61^

That negative controls should be used more routinely in epidemiological studies has been suggested,^1^^,^^8^ and adjustment of the effect or *P*-value of the exposure on the outcome for the negative control has been suggested.^42–45^ A number of the studies described in Table 1 adjust the estimated effect of the exposure for the negative control in order to attempt to account for any unobserved confounding in the model.^23^^,^^25^^,^^31^^,^^34^^,^^36^^,^^39^^,^^46^ As discussed further below, adjusting for the effect of the negative control is not the correct interpretation of negative control exposure studies, as any measurement error in either the exposure or negative control will affect the results obtained from the analysis. The results obtained from the negative control exposure study should instead contribute to a triangulation of evidence from a range of sources which are subject to different sources of potential bias, and feed into the overall result.^1^^,^^2^^,^^11^

One key assumption made in negative control studies is that the relationship between the exposure and outcome and the control and outcome are subject to the same confounding. However, in order to obtain a consistent estimate of the effect of the exposure on the outcome, we also need to make the additional assumption that there is no differential measurement error in either the exposure or the negative control. Measurement error in an explanatory variable in a linear regression will lead to a biased estimate of the association between that variable and the outcome.^47^^,^^48^ Therefore, if there is measurement error in either the exposure or the negative control, the estimated coefficients from the regression of the outcome on the exposure and the negative control will be biased estimates of the true association between these variables and the outcome.

It is likely that the exposure and control variables may be subject to different levels of measurement error. For example, a negative control for the intrauterine effect of a mother’s exposure on their child’s outcome, that is often used, is the father’s exposure. However, the father’s data are likely to suffer from a higher level of measurement error than the mother’s if they are collected from the mother (as they often are), as the data will be less accurately recalled. This potential difference in measurement error is important; if there is a higher level of measurement error in the negative control than in the exposure variable, then the association between the negative control and the outcome may appear to be weak or null even when unmeasured confounding factors that relate to both the exposure and the negative control are present.^49^ In the remainder of this paper, we evaluate the effect of adjusting for a negative control on the identification of a causal effect of the exposure on an outcome when measurement error is present. We adjust for the negative control by examining the difference between the expected value of the coefficient for the exposure and the coefficient for the negative control, in a regression of the outcome on the exposure and the negative control.

## Methods

**Figure 1:**
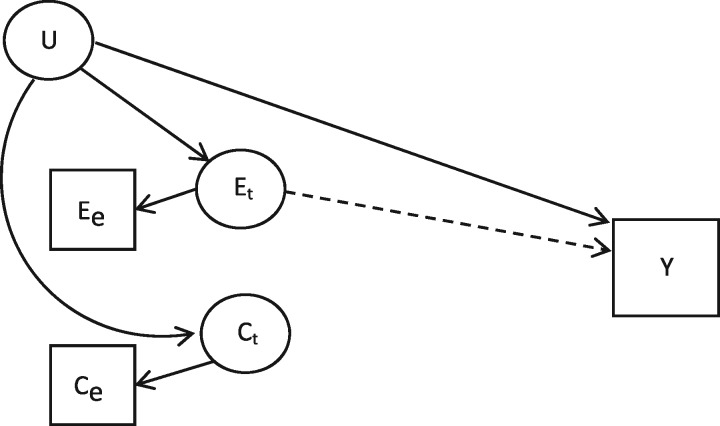
The relationships in an observational negative control exposure study*. *Variables in squares are observed; variables in circles are unobserved.

We examine the effect of measurement error on the interpretation of the estimated coefficients when the association between an exposure and an outcome is interrogated in a negative control exposure study. We consider two examples of negative control exposure studies: first, a model with continuous outcome, exposure and negative control variables; and second, a model with a continuous outcome variable and binary exposure and negative control variables. These models reflect two different scenarios which are frequently found in epidemiological studies, and where negative control exposure methods have previously been used. In each setting, the causal relationship between the exposure, the negative control and the outcome are set up as given in Figure 1. This relationship can be written as:

$$y_{i}=\beta_{1}E_{T,i}+\beta_{2}C_{T,i}+\gamma U_{i}+\varepsilon_{i}$$

$E_{T,i}$ is the true value of the exposure of interest for individual i, $C_{T,i}$ is the true value of the negative control, $U_{i}$ is an unmeasured confounder that is correlated with both the exposure and the control and $y_{i}$ is the outcome. $\varepsilon_{i}$ is a normally distributed random error term with mean 0.

### Continuous exposure and control variables

The exposure and negative control $E_{T,i}$ and $C_{T,i}$ are continuous variables measured with error:

EO,i=ET,i+vE,iCO,i=CT,i+vC,i

where $E_{O,i}$ is the observed value of the exposure for individual i, measured with error, $C_{O,i}$ is the observed value of the negative control for individual i, measured with error, and $v_{E,i}$ and $v_{C,i}$ are normally distributed random error terms with variance $\sigma_{vE}^{2}$ and $\sigma_{vC}^{2}$, respectively. Throughout we assume that this measurement error is uncorrelated with the true values of the exposure and negative control. We additionally make the assumption that $E_{T,i}$ and $C_{T,i}$ are only correlated through the unmeasured confounders $U_{i}$; therefore $\rho_{EC}=\rho_{UE}\rho_{UC}$. In the context of maternal and paternal comparison studies, this assumption implies no assortative mating on the basis of the exposure or control variables. Throughout the analysis, $\rho_{UE}$ and $\rho_{UC}$ are set to 0.4, meaning that $\rho_{EC}$_ _= 0.16. The values of $\sigma_{vE}^{2}$ and $\sigma_{vC}^{2}$ are determined according to the desired values of the intraclass correlation coefficient (ICC), the proportion of total variance in the observed variable that is due to true variation:

$$ICC=\sigma_{u}^{2}/(\sigma_{u}^{2}+\sigma_{v}^{2})$$

$\sigma_{u}^{2}$ is the variance of the true variable and $\sigma_{v}^{2}$ is the variance of the measurement error. An ICC value of 1 indicates that there is no measurement error in the model and $\sigma_{v}^{2}=0$, whereas an ICC value of 0 would indicate that all of the variation in the observed variable was due to the measurement error and $\sigma_{u}^{2}=0$. We derive the bias of the ordinary least squares (OLS) estimator of $\beta_{1}$ and $\beta_{2}$ when the exposure and control are included in the same model. This bias is the difference between the expected value of the estimator and the true value of the parameter. The measurement error in the model will lead to regression dilution bias and so bias the estimated coefficients towards the null; however, the additional presence of an unmeasured confounder that is correlated with the exposure and negative control variables means that the total bias could be towards or away from the null.

In matrix form, the true model considered can be written as:

$$y=X_{T}\beta +U\gamma +\text{ε}$$

where $X_{T}=\left(\begin{array}{cc} E_{T} & C_{T} \end{array}\right)$, $\beta =\left(\begin{array}{c} \beta_{1}\\ \beta_{2} \end{array}\right)$, and $E_{T},C_{T},y,U$ and ε are vectors including all individuals for variables $E_{T,i},C_{T,i},y_{i},U_{i}$ and $\varepsilon_{i}$, respectively. The measurement error can be defined as:

$$X_{e}=X_{t}+V$$

where $X_{O}=\left(\begin{array}{cc} E_{O} & C_{O} \end{array}\right)$ and V $=\left(\begin{array}{cc} v_{E} & v_{C} \end{array}\right),E_{e},C_{e}$, and $v_{E}$ and $v_{C}$ are vectors including all individuals for variables $E_{O,i},C_{O,i}$, $v_{E,i}$ and $v_{C,i}$, respectively. We make the following assumptions about the distribution of the error terms:

(vEvC)∼N(0,Σv)Σv=(σv12σv12σv12σv22)=(σv1200σv22)

and the asymptotic distribution of the variables is given by:

plim1nXT′XT=QXX=(QEEQECQECQCC)=(1ρECρEC1)plim1nXT′U=QXU=(QEUQCU)=(ρEUρCU)

As the confounders U are unobserved, the regression to be estimated is:

(1) $$y_{i}=\beta_{1}E_{O,i}+\beta_{2}C_{O,i}+u_{i}$$

In matrix form this can be written as:

$$y=X_{O}\prime \beta +u$$

In this case it can be shown that the bias of the OLS estimator of β,$\hat{\beta },$ is given by:

(2) E(β^−β)=E((β^1β^2)−(β1β2))=(QXX+Σv)−1QXUγ−(QXX+Σv)−1Σvβ.

The derivation of this is given in Appendix 1, available as Supplementary data at *IJE* online.

Using this equation for the bias, we calculate the bias of ${\hat{\beta }}_{1}$ and the bias of the difference between the coefficients for the exposure and the negative control, i.e. $\left({\hat{\beta }}_{1}-{\hat{\beta }}_{2}\right)$, with and without measurement error in each of the exposure and the negative control and for a range of values ofγ, the effect of the unmeasured confounder on the outcome.

### Binary exposure and control variables

We then consider a model where the exposure and control are both binary variables which take values of 0 or 1. For this set-up, we conducted Monte Carlo simulations to examine the difference between the estimated association and the true effect for $\hat{\beta }$_1_, and for $\left({\hat{\beta }}_{1}-{\hat{\beta }}_{2}\right)$, for different levels of measurement error.

In this scenario, the true values of the exposure and the control, $E_{t}$ and $C_{t}$, were dichotomized into binary variables by classing those observations with the highest 20% of values as 1 and the rest as 0. Measurement error was introduced in this model by reclassifying a proportion of the true values for the exposure and the control, to take the opposite value. This proportion was changed in order to change the level of the measurement error in the model, but was applied equally to the ‘true’ (1) and ‘false’ (0) values. Other than this change, the model was set up in the same way as in the continuous case, and the true relationship between the binary exposure and control variables and the outcome variable is given by:

$$y_{i}=\beta_{1}E_{T,i}+\beta_{2}C_{T,i}+\gamma U_{i}+\varepsilon_{i}$$

As before, the outcome variable in this model is a continuous variable and so the model is estimated using OLS. Simulations were conducted for two scenarios: where neither the exposure or the control have a true effect on the outcome; and where the exposure has an effect on the outcome and the negative control has no effect on the outcome. In each of these scenarios, different levels of measurement error in each of the exposure and the control were considered: no measurement error; a low level of measurement error where 10% of the observations are misclassified; and a high level of measurement error where 50% of the observations are misclassified. 

## Results

### Continuous exposure and control variables

**Figure 2:**
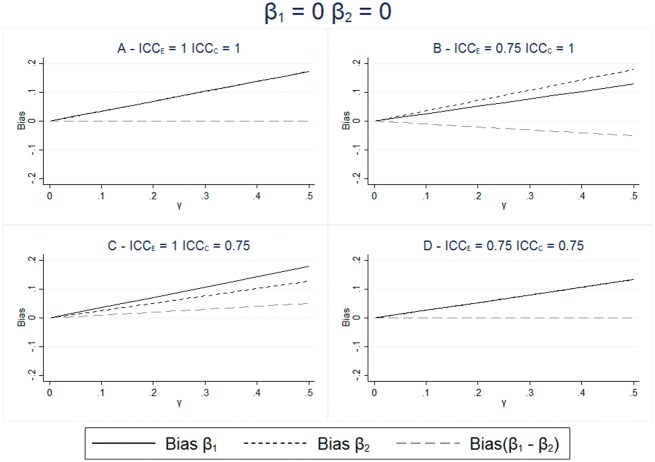
Bias in estimated effect of the exposure and negative control; exposure and negative control each have no effect on the outcome. The bias in the exposure and negative control are calculated from the expression given in equation (2) with and without measurement error in the exposure and negative control. Neither the exposure or the negative control have any effect on the outcome; $\beta_{1}=0,\beta_{2}=0$. The effect of the unmeasured confounding varies between γ=0 and γ=0.5. $\rho_{UE}$_ _= $\rho_{UC}$_ _= 0.4.

**Figure 3:**
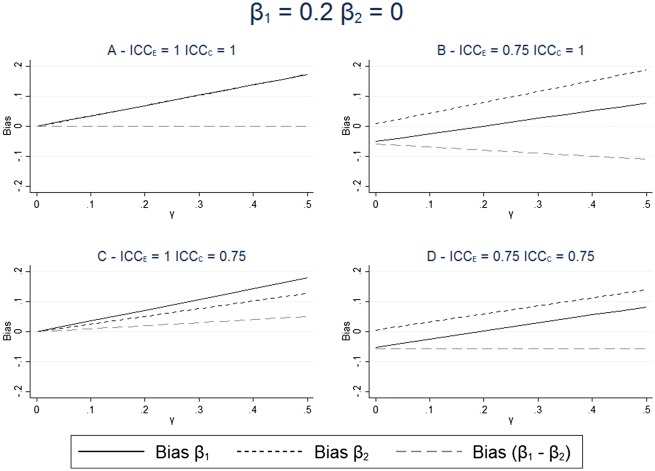
Bias in estimated effect of the exposure and negative control; the exposure has a causal effect on the outcome. The bias in the exposure and negative control are calculated from the expression given in equation (2), with and without measurement error in the exposure and negative control. The exposure has an effect on the outcome: $\beta_{1}=0.2$; the negative control has no effect on the outcome: $\beta_{2}=0$. The effect of the unmeasured confounding varies between γ=0 and γ=0.5. $\rho_{UE}$_ _= $\rho_{UC}$_ _= 0.4.

The results for the bias for different levels of measurement error and effect of the unmeasured confounder are given in Figures 2 and 3. These figures show that in all scenarios where the unmeasured confounder has an effect on the outcome, the estimate of $\beta_{1}$ is not equal to the true effect and the inclusion of the negative control in the model does not remove this difference. These figures also show that when there is no measurement erro, (Figure 2A and Figure 3A), then the estimation of the exposure adjusted for the negative control $\left({\hat{\beta }}_{1}-{\hat{\beta }}_{2}\right),$correctly estimates the causal effect of the exposure on the outcome. However, when there is measurement error, then this estimate is only unbiased when both the measurement error and the effect size are the same for both the exposure and the negative control, as shown in Figure 2D.

These results show that the difference between the estimated effect of the exposure and the estimated effect of the negative control is not equal to the true difference in the causal effect of the two variables, except under a strong set of assumptions. Therefore, we cannot reliably estimate the size of the effect of the exposure on the outcome, when there is measurement error, by looking at the difference in the association between the exposure and the outcome and the negative control and the outcome. Figure 2B and Figure 2C show that differing measurement error in the exposure and the control can lead to different sizes of estimated coefficients for $\beta_{1}$ and $\beta_{2}$, even when neither of the variables has a direct effect on the outcome.

### Binary exposure and control variables

We ran Monte Carlo simulations with a binary exposure and binary control and a continuous outcome variable, for different levels of association between the variables and for different proportions of the exposure and the control misclassified. Table 2 shows the results for a scenario where there is no true association of either the exposure or the negative control with the outcome. Table 3 shows the results where the exposure has a positive effect on the outcome and the negative control has no causal effect on the outcome.

**Table 2:** β_1_ = β_2_ = 0—simulation results for bias in estimated effect of binary exposure and negative control; exposure and negative control each have no effect on the outcome

Error in exposure	Error in negative control	Bias for ${\hat{\beta }}_{1}$	Bias for ${\hat{\beta }}_{2}$	Bias for ${\hat{\beta }}_{1}-{\hat{\beta }}_{2}$
None (0%)	None (0%)	0.140	0.140	0.000
None (0%)	Low (10%)	0.140	0.093	0.046
None (0%)	High (50%)	0.140	0.000	0.140
Low (10%)	None (0%)	0.093	0.140	−0.047
Low (10%)	Low (10%)	0.093	0.093	0.000
Low (10%)	High (50%)	0.093	0.000	0.093
High (50%)	None (0%)	0.000	0.140	−0.140
High (50%)	Low (10%)	0.000	0.093	−0.094
High (50%)	High (50%)	0.000	0.000	0.000

Bias in the estimated values of ${\hat{\beta }}_{1}$, ${\hat{\beta }}_{2}$ and $({\hat{\beta }}_{1}-{\hat{\beta }}_{2}$) when the exposure and negative control variables are binary and the outcome is continuous. Measurement error is the proportion of observations misclassified: $\beta_{1}\;=\;\beta_{2}\;=\;0$. Effect of the unmeasured confounder, γ = 0.2: $\rho_{UE}$_ _= $\rho_{UC}$_ _= 0.4.

**Table 3:** β_1_ = 0.2, β_2_ = 0—simulation results for bias in estimated effect of binary exposure and negative control; exposure has a causal effect on the outcome

Error in exposure	Error in negative control	Bias for ${\hat{\beta }}_{1}$	Bias for ${\hat{\beta }}_{2}$	Bias for ${\hat{\beta }}_{1}-{\hat{\beta }}_{2}$
None (0%)	None (0%)	0.140	0.157	−0.017
None (0%)	Low (10%)	0.140	0.104	0.035
None (0%)	High (50%)	0.140	0.000	0.140
Low (10%)	None (0%)	0.026	0.157	−0.131
Low (10%)	Low (10%)	0.026	0.104	−0.078
Low (10%)	High (50%)	0.026	0.000	0.026
High (50%)	None (0%)	−0.200	0.157	−0.357
High (50%)	Low (10%)	−0.200	0.104	−0.305
High (50%)	High (50%)	−0.200	0.000	−0.200

Bias in the estimated values of ${\hat{\beta }}_{1}$, ${\hat{\beta }}_{2}$ and $({\hat{\beta }}_{1}-{\hat{\beta }}_{2}$) when the exposure and negative control variables are binary and the outcome is continuous. Measurement error is the proportion of observations misclassified: $\beta_{1}\;=\;0.2,\beta_{2}\;=\;0$. Effect of the unmeasured confounder, γ = 0.2: $\rho_{UE}$_ _= $\rho_{UC}$_ _= 0.4.

The results here show the same pattern as the analytical results for the continuous example and show that, in all of the scenarios considered, the effect of $\left({\hat{\beta }}_{1}-{\hat{\beta }}_{2}\right)$ is only unbiased when the effects of the exposure and of the negative control on the outcome are the same, and either there is no measurement error in the model or the measurement errors for each of the exposure and the negative control are the same. These results additionally show that when the level of misclassification is high, ${\hat{\beta }}_{1}$is always zero, leading to a large difference between ${\hat{\beta }}_{1}$ and $\beta_{1}$ when $\beta_{1}$ is large but no difference when $\beta_{1}$is zero.

## Discussion

Due to the unmeasured confounding that is inherent in studies in which it is necessary to use a negative control, the estimates of regression coefficients are always expected to reflect the confounded association rather than the causal relationship. In the analysis above, we have shown that measurement error in the exposure and negative control will add a bias which may increase or decrease the difference between the estimated coefficient and the causal relationship. The implication of this is that adjusting the estimated effect of the exposure for the estimated effect of the negative control variable, as suggested as a way to account for the bias created by confounding^42–45^ and implemented in a number of studies described in Table 1,^23^^,^^25^^,^^31^^,^^34^^,^^36^^,^^39^^,^^46^ does not necessarily improve our estimates of the associations between the exposure and the outcome. This indicates that the results from a negative control study cannot be used to estimate the size of a causal effect directly, but instead can feed into a triangulation of evidence which is subject to different sources of bias, to strengthen evidence regarding whether or not a causal effect of the exposure on the outcome is present.^6^^,^^13^ In cases where measurement error in the exposure and negative control variables is likely to be minimal, such as where they are germline genetic variants, the total error in the estimate of the effect of the exposure adjusted for the negative control will be small. However, this will only be the case if it is known that the measurement error in both the exposure and negative control is minimal, and so will only apply to a very small proportion of studies. Control outcome calibration is a method which has been proposed to estimate the size of an effect of an exposure in a negative control outcome study, even when the outcome of interest and control outcome do not have the same scale,^50–52^ and has been suggested for negative control exposure studies.^50^ The results given here show that any calibration based on the results from a negative control study should be used with caution. The results presented here also show that such calibration methods will not transfer directly to models with a negative control exposure variable as measurement error in either the exposure, or the negative control will distort the obtained results.

Throughout this analysis we have assumed that there are no measured confounders in the model. The inclusion of measured confounders that were not subject to any measurement error would not change the results in the analysis above. However, as has been discussed previously,^53^ the addition of measured confounders to the model, which are also measured with error, will lead to a more complex bias in the estimated effects of the exposure on the outcome. The addition of such variables, however, would not change the overall conclusion that caution should be used when interpreting results from negative control studies, as any additional confounders will add to the potential for the results obtained to be very different from the true effect of the exposure on the outcome. We have also assumed no direct correlation between the true values of the exposure and control; however, relaxing this assumption will also not change the conclusions that can be drawn from the results.

The results presented above show that negative controls can be useful in contributing to the triangulation of evidence regarding whether or not a causal relationship is likely to exist between an exposure and outcome of interest.^7^ However, negative control exposure studies should not be used to obtain a point estimate of the causal effect of the exposure of interest on the outcome as, in the presence of measurement error in either the exposure or the control, this effect estimate will also be subject to bias. The results we have found mean we cannot give a general statement about the direction of any bias caused by measurement error in a negative control exposure study. 

## Supplementary Data

Supplementary data are available at *IJE* online.

## Funding

This work was supported by the Medical Research Council and the University of Bristol [MRC Integrative Epidemiology Unit (IEU) MC_UU_12013/1 and MC_UU_12013/9].

**Conflict of interest:** None declared.

## Supplementary Material

Supplementary Data
