# Supplementary material for: Negative control exposure studies in the presence of measurement error: implications for attempted effect estimate calibration
Source: Int J Epidemiol. 2017 Oct 27;47(2):587–96. doi: 10.1093/ije/dyx213 (PMC5913619; doi:10.1093/ije/dyx213)
Supplement: Supplementary Data [file dyx213_supp.pdf]

Appendix 1

In matrix form the true model considered can be written as;

$$\boldsymbol{y} = \boldsymbol{X_T\beta} + \boldsymbol{U}\gamma + \boldsymbol{\epsilon}$$

The measurement error can be defined as;

$$\boldsymbol{X}_O = \boldsymbol{X}_T + \boldsymbol{V}$$

Where the variables and error terms are defined as given in the body of the paper.

In matrix form the regression estimated can be written as;

$$\boldsymbol{y} = \boldsymbol{X}'_O\boldsymbol{\beta} + \boldsymbol{u}$$

The OLS estimator for $\boldsymbol{\beta}$ is given by;

$$\widehat{\boldsymbol{\beta}}_{OLS} = (\boldsymbol{X}'_O\boldsymbol{X}_O)^{-1}(\boldsymbol{X}'_O\boldsymbol{y})$$

$$= (\boldsymbol{X}'_O\boldsymbol{X}_O)^{-1}\big(\boldsymbol{X}'_O(\boldsymbol{X}_O\boldsymbol{\beta} + \boldsymbol{U}\gamma - \boldsymbol{V\beta} + \boldsymbol{\epsilon})\big)$$

$$= \boldsymbol{\beta} + (\boldsymbol{X}'_O\boldsymbol{X}_O)^{-1}(\boldsymbol{X}'_O\boldsymbol{U}\gamma) - (\boldsymbol{X}'_O\boldsymbol{X}_O)^{-1}(\boldsymbol{X}'_O\boldsymbol{V\beta}) + (\boldsymbol{X}'_O\boldsymbol{X}_O)^{-1}(\boldsymbol{X}'_O\boldsymbol{\epsilon})$$

Therefore the bias of $\widehat{\boldsymbol{\beta}}_{OLS}$ is given by;

$$E(\widehat{\boldsymbol{\beta}}_{OLS}) - \boldsymbol{\beta} = (\boldsymbol{X}'_O\boldsymbol{X}_O)^{-1}(\boldsymbol{X}'_O\boldsymbol{U}\gamma) - (\boldsymbol{X}'_O\boldsymbol{X}_O)^{-1}(\boldsymbol{X}'_O\boldsymbol{V\beta}) + (\boldsymbol{X}'_O\boldsymbol{X}_O)^{-1}(\boldsymbol{X}'_O\boldsymbol{\epsilon})$$

The asymptotic distribution of this bias can be found by taking the probability limit of this expression;

$$plim\frac{1}{n}(\boldsymbol{X}'_O\boldsymbol{X}_O) = plim\frac{1}{n}(\boldsymbol{X}'_T\boldsymbol{X}_T + 2\boldsymbol{X}'_T\boldsymbol{V} + \boldsymbol{V}'\boldsymbol{V}) = \boldsymbol{Q}_{XX} + 2\boldsymbol{\Sigma}_{XV} + \boldsymbol{\Sigma}_V plim\frac{1}{n}(\boldsymbol{X}'_O U)$$

$$= plim\frac{1}{n}(\boldsymbol{X}'_T U + \boldsymbol{V}'U) = \boldsymbol{Q}_{XU} + 0$$

$$plim\frac{1}{n}(\boldsymbol{X}'_O\boldsymbol{V}) = plim\frac{1}{n}(\boldsymbol{X}'_T\boldsymbol{V} + \boldsymbol{V}'\boldsymbol{V}) = \boldsymbol{\Sigma}_{XV} + \boldsymbol{\Sigma}_V$$

$$plim\tfrac{1}{n}(\boldsymbol{X}'_O\epsilon) = plim\tfrac{1}{n}(\boldsymbol{X}'_T\epsilon + \boldsymbol{V}'\epsilon) = 0 + 0$$

Therefore the bias of the OLS estimator of $\boldsymbol{\beta}$ is;

$$E(\widehat{\boldsymbol{\beta}}_{OLS}) - \boldsymbol{\beta} \overset{d}{\rightarrow} (\boldsymbol{Q}_{XX} + 2\boldsymbol{\Sigma}_{XV} + \boldsymbol{\Sigma}_V)^{-1}\boldsymbol{Q}_{XU}\gamma - (\boldsymbol{Q}_{XX} + 2\boldsymbol{\Sigma}_{XV} + \boldsymbol{\Sigma}_V)^{-1}(\boldsymbol{\Sigma}_{XV} + \boldsymbol{\Sigma}_V)\boldsymbol{\beta}$$

$$+(\boldsymbol{Q}_{XX} + 2\boldsymbol{\Sigma}_{XV} + \boldsymbol{\Sigma}_V)^{-1}(0)$$

$$= (\boldsymbol{Q}_{XX} + 2\boldsymbol{\Sigma}_{XV} + \boldsymbol{\Sigma}_V)^{-1}\boldsymbol{Q}_{XU}\gamma - (\boldsymbol{Q}_{XX} + 2\boldsymbol{\Sigma}_{XV} + \boldsymbol{\Sigma}_V)^{-1}(\boldsymbol{\Sigma}_{XV} + \boldsymbol{\Sigma}_V)\boldsymbol{\beta}$$

As we have assumed no correlation between the true values of the exposure and control and the error terms in the model this can be simplified to;

$$E(\widehat{\boldsymbol{\beta}}_{OLS}) - \boldsymbol{\beta} = (\boldsymbol{Q}_{XX} + \boldsymbol{\Sigma}_V)^{-1}\boldsymbol{Q}_{XU}\gamma - (\boldsymbol{Q}_{XX} + \boldsymbol{\Sigma}_V)^{-1}\,\boldsymbol{\Sigma}_V\boldsymbol{\beta}.$$
